# Reading and Myopia: Contrast Polarity Matters

**DOI:** 10.1038/s41598-018-28904-x

**Published:** 2018-07-18

**Authors:** Andrea C. Aleman, Min Wang, Frank Schaeffel

**Affiliations:** Section of Neurobiology of the Eye, Ophthalmic Research Institute, University of Tuebingen, Tuebingen, Germany

## Abstract

In myopia the eye grows too long, generating poorly focused retinal images when people try to look at a distance. Myopia is tightly linked to the educational status and is on the rise worldwide. It is still not clear which kind of visual experience stimulates eye growth in children and students when they study. We propose a new and perhaps unexpected reason. Work in animal models has shown that selective activation of ON or OFF pathways has also selective effects on eye growth. This is likely to be true also in humans. Using custom-developed software to process video frames of the visual environment in realtime we quantified relative ON and OFF stimulus strengths. We found that ON and OFF inputs were largely balanced in natural environments. However, black text on white paper heavily overstimulated retinal OFF pathways. Conversely, white text on black paper overstimulated ON pathways. Using optical coherence tomography (OCT) in young human subjects, we found that the choroid, the heavily perfused layer behind the retina in the eye, becomes about 16 µm thinner in only one hour when subjects read black text on white background but about 10 µm thicker when they read white text from black background. Studies both in animal models and in humans have shown that thinner choroids are associated with myopia development and thicker choroids with myopia inhibition. Therefore, reading white text from a black screen or tablet may be a way to inhibit myopia, while conventional black text on white background may stimulate myopia.

## Introduction

Myopia became recently the most frequent vision disorder in young people. At present, about 50 percent of university students are myopic in central Europe^1,2^, but the numbers are considerably higher in Taiwan, Singapore, Korea, Japan and in the large cities of China^3^. It has been predicted that half of the world population will be myopic by the year 2050^4^. While low myopia (less than 5 D) is merely disturbing, requiring spectacle or contact lens correction, higher myopia is associated with an increasing risk of retino-choroidal degeneration, glaucoma and cataract, causing a significant risk of blindness already in the middle of the life span^5^. There is urgent need to slow myopia progression in young people to avoid it reaching critical levels^6^. Current attempts include novel optical corrections (multifocal contact lens designs^7^), as well as refractive gradient lens designs^8^, more outdoor activity before school age^6^, or low dose atropine eye drops^9^. However, there is agreement that it would be much better to prevent myopia from beginning^10^.

There is solid evidence that myopia is associated with the level of education^1,2,6^. Each year of study has been found to move the average refraction in the myopic direction by about 0.5 diopters^11^. Traditionally, reading and near work have been associated with myopia onset and progression^12^. However, it is still not clear how exactly the visual input looks like that may drive myopia during reading. Previous attempts to explain the link between reading and myopia have largely failed. A promising candidate was the “lag of accommodation”^13^. Since people tend to accommodate too little when they read, the best focused image may be behind the retina. Experiments in animal models have shown that this condition stimulates eye growth^14^ as the retina tries to “catch the focus”. However, in children it was found that the “lag of accommodation” develops concomitantly with myopia^15^, and not before, reducing the probability of a causal relationship.

In this study, we provide theoretical and experimental evidence for a new and unexpected factor that may drive myopia development during reading. The visual system is organized into ON and OFF pathways. Retinal ganglion cells have circular fields that are organized into center ON/periphery OFF-structures, or vice versa^16^. An important consequence is that homogenously illuminated regions in the visual field do not excite the ganglion cells, avoiding that unnecessary visual information (like absolute pixel luminance) is transmitted to the visual cortex. Small ON or OFF receptive field sizes, like in the foveal region, generate high sensitivity to fine details since they respond best to spatial contrast modulation at the highest detectable Fourier components in the retinal image. There are clear functional differences between ON and OFF pathways. After monkeys was given D,L-2-amino-4-phosphonobutyric acid (APB), a glutamate agonist and highly selective ON-channel blocker, they could no longer recognize small targets that were shown against a darker background^17^. Obviously, contrast sensitivity is strongly reduced when the ON channel was blocked^18^. On the other hand, the OFF pathway mediates higher spatial resolution. In guinea pig retina, OFF retinal ganglion cells that respond to dark spots on bright background (negative contrast) have smaller dendritic fields, therefore also smaller receptive fields, and are about twice as numerous as ON cells that respond to positive contrast^19^.

ON and OFF pathways have selective effects on eye growth and myopia. In 1991, Smith *et al*. found that ON pathway blockade by APB reduced ocular growth and induced hyperopia in cats^20^ and Crewther et al obtained similar results chickens^21^. Animal models can be made myopic when they wear negative spectacle lenses for some time, and hyperopic with positive lenses^22^. In 2002, Crewther and Crewther^23^ discovered that chickens become less myopic with negative lenses when they were stimulated with sawtooth-shaped temporal luminance profiles, i.e. rapid increases in luminances with slow decay. With the reversed stimulus profiles, they became less hyperopic when they wore positive lenses. Later, Crewther and Crewther^24^ showed that the gliotoxin L-α-aminoadipic acid (LAA) which eliminates the ON response in the electroretinogram (ERG), also reduced negative lens induced myopia, while D-α-aminoadipic acid (DAA) which suppressed the OFF response in the ERG, reduced hyperopia induced by positive lenses. Findings in transgenic mice were generally in line with the findings from pharmacological inactivation. Knock-out mice mutants lacking functional ON channels (nob −/−; mGluR6 −/−) became more myopic when they wore diffusers over their eyes^25,26^ while mutants lacking functional OFF channels (vsx1 −/− mice) developed similar amounts of deprivation myopia as wildtype^27^. Taken together, there is considerable evidence that there are selective effects of ON and OFF pathway activation on eye growth and refractive error development.

It is not known whether predominant ON or OFF stimulation might also affect myopia development in humans. The relative signal strength in the two channels would be important to know in various visual environments and could be related to myopia development. Natural scenes have previously been analyzed, for instance, by Ratliff *et al*.^19^. They found that leaves on the ground have more negative contrast which would predominantly stimulate OFF ganglion cells. They concluded that the higher abundancy of OFF retinal ganglion cells in guinea pig retina represents an adaptation to the more abundant negative contrasts in their visual environment, supporting their hypothesis that there are “equal synapses for equal bits”. We have written software in Visual C++ for realtime analysis of monochrome video movies (640 × 480 pixels) in terms of ON and OFF contributions, to quantify the relative ON and OFF input strength in typical visual environments of humans. We have related the relative ON and OFF input strength to changes in the thickness of the choroid, the heavily perfused layer behind the retina. Choroidal thickness changes are known to be associated with subsequent changes in eye growth and emmetropization^28–34^.

## Results

### Relative input strength of ON and OFF pathways during reading

**Figure 1:**
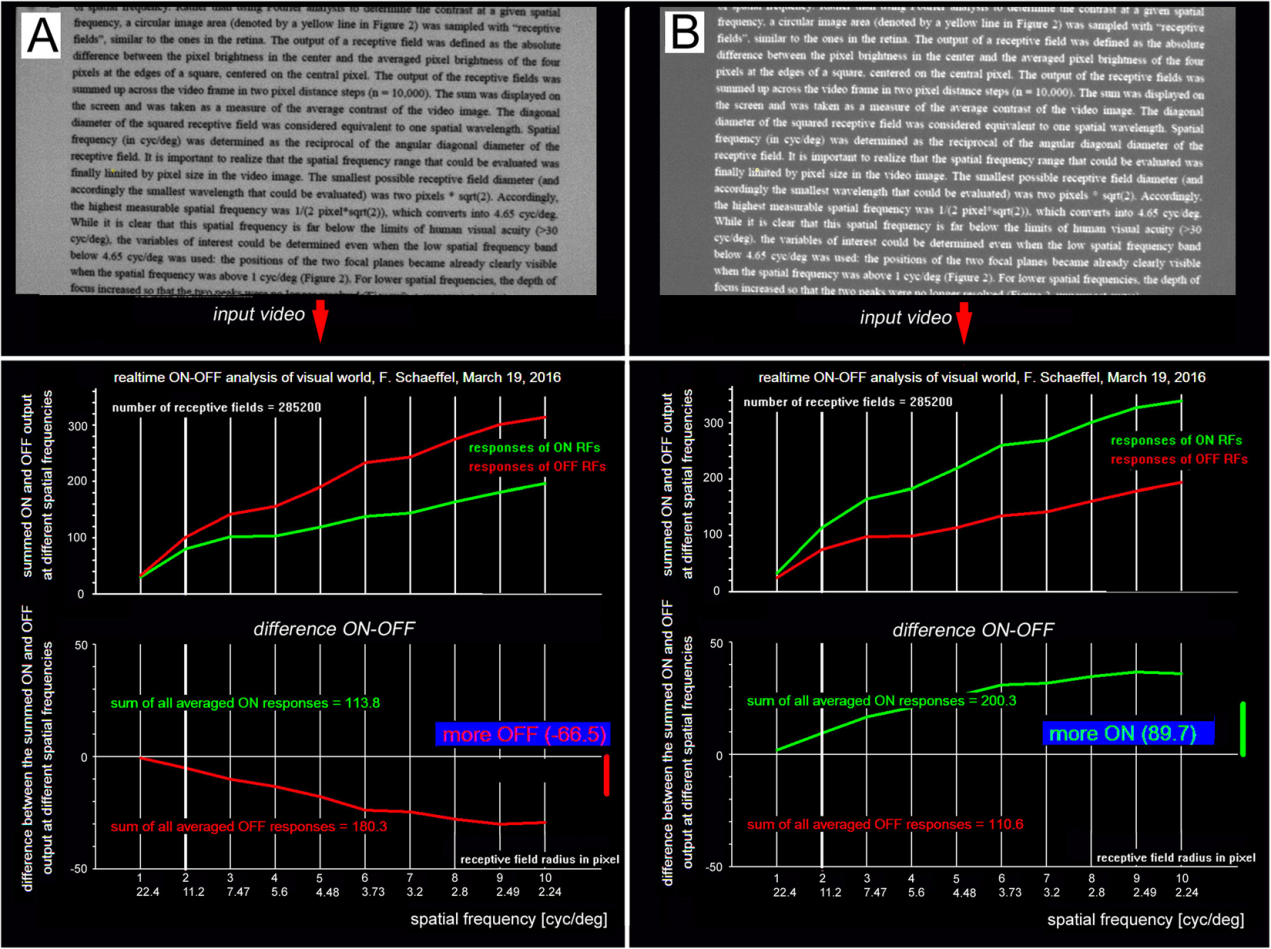
Analysis of the relative strength of ON and OFF stimulation when looking at (**A**) dark text on bright background or (**B**) bright text on dark background. The average luminance of both pictures was matched (top). On the bottom, the output of the software is shown. Green lines indicate the relative strength of ON stimulation, red lines of OFF stimulation, plotted over a range of spatial frequencies (from 22.4 to 2.24 cyc/deg when measured with a 16 mm camera lens). Note that dark text on bright background always overstimulates OFF pathways while bright text on dark overstimulates ON pathways.

Using our software, we found that ON and OFF input strength was largely balanced in different natural scenes (outdoors, but also in the building; see example in the Methods section). However, black text on white background represented a severe overstimulation of the OFF channels while white text on black background overstimulated the ON channels (Fig. 1). This was true for both Latin and Chinese characters. To exclude that this finding may be confounded by non-linearities in the video system, we tested how stable the asymmetry in ON or OFF stimulation was against changes in video image brightness. The asymmetry persisted from low pixel values to close to saturation (average pixel values in the image between 20 to 240). Nevertheless, to exclude confounding effects of absolute brightness, the average brightness was matched for black text on light background or white text on dark background in our analysis (Fig. 1A,B).

### Changes in choroidal thickness induced by reading text with different contrast polarity

**Figure 2:**
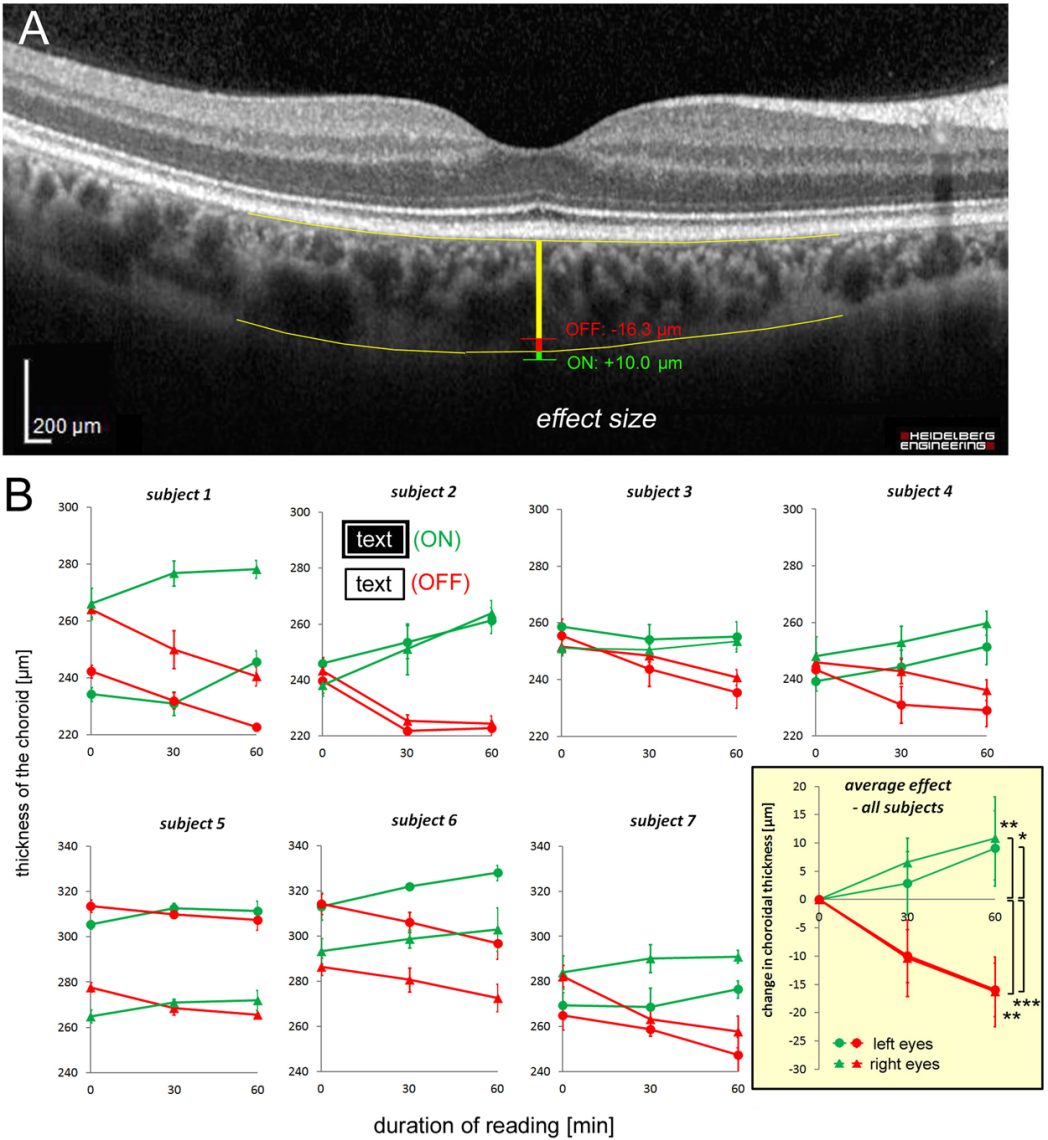
(**A**) Sample picture of the foveal area, obtained from the Spectralis OCT. The measurement procedure to determine subfoveal choroidal thickness is illustrated (yellow bar, between the two yellow lines; see also the methods section), together with the average effect size observed in the current study. (**B**) Absolute choroidal thickness in both eyes of the seven subjects is shown at the beginning of the reading task, and after 30 and 60 minutes. Box in yellow: average effects in all subjects. Note that reading white text on black background (ON stimulus, denoted in green) causes choroidal thickening while black text on white background caused choroidal thinning (OFF stimulus, denoted in red). Triangles denote right eyes, circles left eyes. Error bars are standard deviations. Significance levels after 60 minutes: ***p < 0.001, **p < 0.01, *p < 0.05 (two-sided t-tests, no multiple comparisons).

In the next step, we tested whether a well known precursor of eye growth during emmetropization, the thickness of the choroid (Fig. 2A), may change in human subjects when they were reading black text on white or white text on black for one hour.

Strikingly, in all seven tested subjects, the choroid became significantly thicker in both eyes (p < 0.05 or better in all cases, paired t-tests) when they read white text on black and significantly thinner (p < 0.01) when they read black text on white background. The average effect in all subjects can be seen in the bottom right in the yellow box (Fig. 2B). Reading black text on white background induced choroidal thinning after one hour by −16.13 ± 4.54 µm (two-sided paired t-test, p = 8.3 * 10^−5^, if both eyes were pooled; right eyes: −16.25 ± 6.16 µm, p = 0.00043, left eyes −15.93 + 4.69 µm, p = 0.00011) while reading white text on black made the choroid thicker by +9.96 ± 6.51 µm (p = 0.0067, if both eyes were pooled; right eyes +10.84 ± 7.30 µm, p = 0.008; left eyes +9.07 ± 6.65, p = 0.011). Further analyses with repeated ANOVAs, followed by t-tests, showed that the changes in the right eyes with ON stimulation were significant both after 30 min (p = 0.02) and 60 min (p = 0.0003). With OFF stimulation, the significance levels were p = 0.0005 and p < 0.0001, respectively. In the left eyes, the values were p = 0.2 and 0.0082 for ON stimulation, and p < 0.0001 and p < 0.0001 for OFF stimulation. We also found that choroidal thinning during OFF stimulation was more pronounced in myopic subjects (amount of choroidal thinning [µm] = 1.44 *refractive error - 11.8; R = 0.71, p < 0.001, when both eyes were considered independent^35^), while choroidal thickening with ON stimulation was not correlated with refractive errors.

## Discussion

We found that reading dark text on bright background reduces choroidal thickness in one hour, while reading bright text on dark background increases the thickness of the choroid. Since choroidal thickness changes are precursors for future changes in eye growth, we expect that there will be selective effects on subsequent myopia development. However, there remain a few key questions:

1. Is it the ON or the OFF pathway which triggers thinner choroids and therefore potentially myopia? Our software showed that bright text on dark background is ON dominated. It induced choroidal thickening, assumed to inhibit myopia. Also Crewther and Crewther (2002)^23^ found that light stimuli with a fast brightness onset (a classical ON stimulus in the ERG) inhibit myopia in the chicken. Surprisingly, pharmacological inhibition of the ON pathway also reduces myopia^20,24^ while genetic inactivation increases it^25,26^. Similarly, both visual OFF stimulation and pharmacological inhibition of the OFF pathway cause relatively more myopia^20,24^ while genetic inactivation is associated with less deprivation myopia^27^. The apparent contradiction could be resolved by assuming that prolonged visual stimulation of the ON or OFF pathways reduces their relative input strength due to adaptation, finally generating similar effects as pharmacological inhibition of the respective pathway. If this would be true, it would in fact be the relatively enhanced OFF input that inhibits myopia. However, more data are needed to confirm this conclusion. One possible approach would be to compare retinal dopamine release after ON or OFF stimulation in animal models since dopamine is well known as an inhibitor of myopia in various animal models^36^.
2. Why does text polarity determine the input strength for the ON and OFF pathways? The software provides a clear answer but it would be satisfying to understand the result intuitively. Black text on white paper contains large bright areas with constant luminance. Neither ON nor OFF receptive fields would provide any output in these areas. However, on the black lines of the letters, most receptive field responses will be OFF, because the dark center pixels of the receptive fields are surrounded by, on average, brighter pixels, generating negative contrast. If the output of all receptive fields is added, the overall result is “OFF dominance”. The opposite is true for bright text on dark background. In general, the closer the ratio of bright to dark areas is to “one”, the more similar is ON and OFF stimulation. We verified that very thick black letters on white paper are less OFF stimulating than thin letters which are the conventional mode. Ratliff *et al*.^19^ describe more negative contrasts in their analyses of photographs of natural scenes. Their analysis was based on more complex simulated receptive field structures, consisting of normalized difference of Gaussian filters. Our receptive fields were more simple but we believe that they describe nevertheless the relative ON/OFF stimulus strength. At least, the output of our software is plausible.
3. How could ON or OFF stimulation affect choroidal thickness? Choroidal thickness was proposed to be an indicator of future changes in axial eye growth in children^28,37^. It was first found in chickens that the choroid thickens when they develop hyperopia or recover from induced myopia while it thins when myopia develops^30^. Later, choroidal thickness changes were also found in marmosets^31^ and rhesus monkeys^32^ with induced refractive errors. Choroidal thickness changes may be considered as an attempt of the eye to reduce image defocus on the retina, although they are too small in human eyes for a significant optical improvement. The mechanisms by which the choroid controls scleral growth rates are not clear. Three hypotheses were proposed^33^ (1) signals from the retina and RPE may trigger the choroid to release growth factors that modulate scleral growth, (2) the thickness of the choroid might determine the diffusion of retinal signals to control scleral growth, an idea that received support by the observation that thicker choroids predict less eye growth and (3) thicker choroids might mechanically reduce the pressure on the sclera, reducing its stretching and growth.
4. Could other factors like stress when the subject has to read text with inverted contrast, or potentially different pupil sizes change choroidal blood flow and thereby its thickness? Is there a chance that the choroidal effects have nothing to do with ON /OFF stimulation? We used no objective measure to determine stress levels when our subjects were asked to read text with inverted contrast so that this question cannot be answered. However, pupil sizes were checked in all subjects. On average, pupils were slightly larger with the darker background, despite similar average brightness, but this effect was not significant. We have tested one subject (#4) with an empty screen at 3 different screen luminances (35, 48, and 62 cd/m², see Methods). Without text, no changes in choroidal thickness were detected. Obviously, the choroidal effects were linked to the text display and it seems very unlikely that they were not controlled by the retina. However, even if the retina would analyze other (yet not identified) image features than ON and OFF stimulus strengths, our finding that the choroid displays bi-directional thickness changes when text is read with different contrast polarity would still be applicable.
5. What are the implications of our study? In 2010, Scott Read^35^ and his colleagues found that optical axial length becomes shorter after one hour when young human subjects wore positive lenses, and longer when they wore negative lenses. Positive lenses also increase and negative lenses decrease choroidal thickness in children in two hours^28^. It is common to all these studies that optical defocus was involved to induce choroidal thickness changes. However, under everyday life conditions, defocus is hard to control, given that accommodation level and viewing distances change continuously. In the current study, bi-directional changes in choroidal thickness were visually induced for the first time without imposing signed defocus. We have excluded the possibility that text with different contrast polarity affects the tonus of accommodation. Using infrared photorefraction, we measured accommodation and pupil sizes in four young subjects, when they were reading brightness-matched text of different contrast polarities at 30 cm distance. However, no differences were found. This may open a new area of research, offering the opportunity to inhibit myopia during reading without any changes in focus. The striking effects of contrast polarity suggest that it may not be advisable to read black text on white background. In fact, it appears that this condition represents a risk factor for myopia. The observed effects on choroidal thickness were consistent, and in a similar range as in previous studies with imposed defocus (choroidal thickness changes below 7 µm in children with −3 or +3D spectacle lenses^28^; 20 µm in young adults with −2 or +2D lenses^29^) or with pharmacological intervention (2% homatropine hydrobromide: +14 µm^37^). Read *et al*.^34^ found a significant association between the change in choroidal thickness and the change in axial length over time. Children showing faster axial eye growth exhibited significantly less choroidal thickening over time compared with children showing slower axial eye growth. Consequently, our data suggest that reading text with inverted contrast may be a simple and powerful way to inhibit myopia, perhaps even without the need of reducing reading hours. However, it is clear that an epidemiological study needs to be done in children in the future to confirm the validity of the proposed strategy.

## Methods

### Analysis of the visual world in terms on ON and OFF stimulus strength

**Figure 3:**
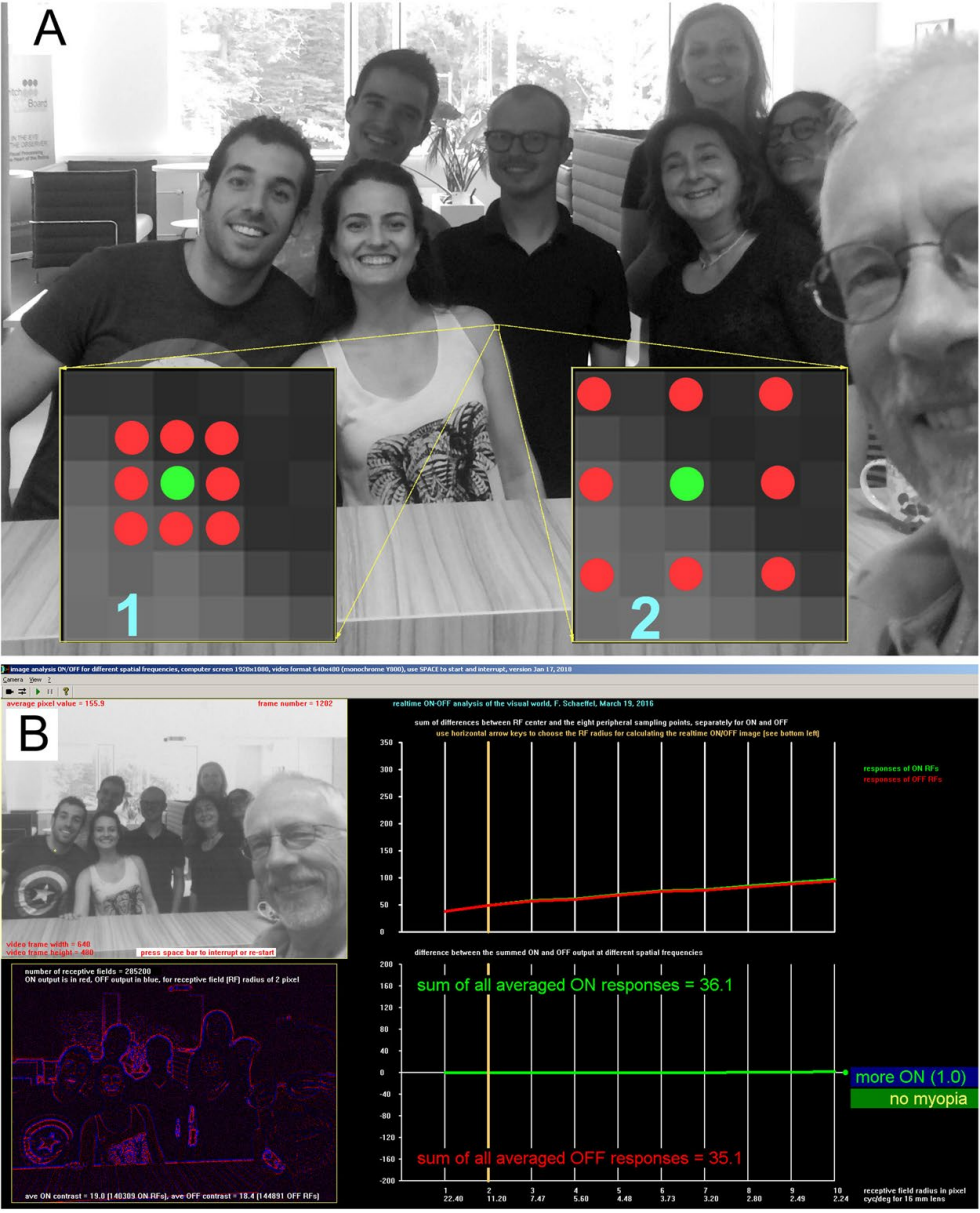
(**A**) Procedure to determine the relative ON and OFF input strength in a scene. Software scanned the video with 285,200 “receptive fields” spaced at regular intervals across the picture. Receptive fields had a simple structure as illustrated by the arrays of green and red dots. The average gray level of 8 peripheral pixels (red) was subtracted from the grey level of the center pixel (green). If the result was positive, the position in the image was considered ON stimulating, if negative, OFF stimulating. The analysis was done simultaneously for different receptive field sizes, and accordingly at different spatial frequencies. The highest sampled spatial frequency by receptive field “1” was about 22.4 cyc/deg with a 16 mm camera lens. Receptive field “2” sampled about 11.2 cyc/deg. The lowest sampled spatial frequency was 2.24 cyc/deg, with the peripheral pixels at a distance of 10 pixels. (**B**) Output (screenshot) of the software. The bottom left picture shows ON outputs in red, OFF outputs in blue. On the right, the sum of all ON and OFF outputs is shown for different spatial frequencies. For the picture above, the sum of all ON and OFF responses were very similar.

At 285,200 regularly spaced positions in the video frames, the gray levels of surrounding pixels were simply subtracted from the gray level of the center pixel. If positive, the position in the image area was considered ON stimulating and if negative, OFF stimulating. The analysis was done for different receptive field sizes, to read out the strength of ON and OFF stimulation at different spatial frequencies. The output of all receptive fields was linearly summed up and provided numbers reflecting the relative ON or OFF input strength for the analyzed visual environment (Fig. 3). The procedure works in realtime, and 60 Hz video frame rate are no problem for a conventional PC.

### Measurements of choroidal thickness before, during and after reading texts with different contrast polarity

Four important factors need to be considered: (1) Since the expected changes in choroidal thickness were small, potential further changes in the biometry of the eye due to accommodation should be avoided^38^. Indeed, our initial experiments showed clear increases in lens thickness and position, as well as changes in choroidal thickness, when subjects were reading from a computer screen at close distance. Therefore, a large 65″ 4 K TV screen (SONY LED-TV KD 65XD7505) was used, placed at 3.2 m (0.3 D) distance. Capital letter height was 10.3 mm, equivalent to 11.8 minutes of arc. (2) To avoid that effects may be based on luminance differences of the screen, the reading targets were matched in brightness. The average screen luminance was adjusted to about 35 cd/m² in both cases, as measured with a Minolta luminance meter (LS-100, Minolta Camera Co., LTD, Japan). Room lights were switched off but some light entered through the window. (3) To exclude that effects in the eyes were just due to the experimental situation, one subject (#4) was asked to look at the empty screen for one hour, first with 64 cd/m² on the first day, then with 48 cd/m² on the second day, and with 35 cd/m² on the third. No significant changes were observed in the choroid. After 60 min at 62 cd/m², the choroid in the right eye changed by +1.03 ± 7.44 µm, and by −0.64 ± 8.08 µm in the left. At 48 cd/m², the changes were +2.57 ± 5.74 µm and −6.80 ± 10.42 µm, and at 35 cd/m², they were +3.08 ± 8.83 and −2.83 ± 5.14 µm. (4) Since it is known that choroidal thickness varies over the day with an amplitude of up to 30 µm^39,40^, all measurements were done at the same time every morning between 9:55 and 11:00 a.m.

Before subjects started to read, and after 30 and 60 minutes of reading, subfoveal choroidal thickness was measured using Spectral Domain Optical Coherence Tomography (HRA + OCT Spectralis, SN 10543, 04/2016, Heidelberg Engineering; resolution mode: high speed, scan angle: 30 degrees, scan type: B-scan, X-axis 768 pixels, Y-axis 496 pixels, line scan, eye tracking not engaged, scan rate of the live image 8.8 frames/sec). Focus was adjusted for each subject depending on their spherical equivalent refraction.

Measurements of choroidal thickness were done manually by carefully estimating the egdes of the choroid on both sides in the original images provided by the Spectralis, using the public software ImageJ (https://imagej.nih.gov/ij/). Segmented lines were drawn into the original image files where the choroidal borders were located, by extrapolating over 100–200 µm below the subfoveal region. Distances between the lines were measured under the foveal pit also using ImageJ. Brightness of the pictures was adjusted to make the borders of the subfoveal choroid most clearly visible. Since the borders of the choroid were not very well defined (Fig. 2A), we took a number of precautions to ensure that the measurements were reliable. All measurements were repeated at least 5 times and the standard deviations of repeated measurements were determined. They were between 5 and 10 µm, similar to in previous studies by other authors^28,29,39,40^. Furthermore, all authors performed measurements in the original pictures and inter-observer correlations were determined. Inter-observer correlation coefficients ranged between R = 0.764 and 0.991. Furthermore, two authors re-analyzed OCT images that were collected at an earlier time, to determine intra-observer variance. One author had an average absolute difference between repeated measurements of 2.5 percent (average SFChT 249.5 µm, absolute average difference to previous measurement 6.35 µm), the other had an average of 3.0 percent (average SFChT 261.0 µm, absolute average difference to previous measurements 7.8 µm). The squared correlation coefficients for the repeated analyses were 0.899 and 0.910, indicating that about 90 percent of the variance in choroidal thickness was captured in the repeated measurements. In summary, while the effects of reading on choroidal thickness were small, our statistical analyses show that they were clearly significant.

### Subjects

Seven young adult subjects participated, subject 1 was Asian, the others Caucasian, subjects 3 and 6 male, subject 3 wore contact lenses, subjects 1, 2, and 7 spectacles. Ages ranged from 23 to 29 years. Subjects had no known ocular pathologies other than mild or moderate myopia. Three were emmetropic (spherical equivalent (SE) between +0.25 and −0.75 D) and 4 were myopic (SE between −3.50 and −6.25D). All subjects were reading with their habitual refractive error corrections. Informed consent was obtained from all subjects prior to the experiments. Furthermore, informed consent was obtained from the people in Fig. 3 to publish their faces in the online open-access publication. Some of the people in Fig. 3 were also subjects in the study.

### Ethics approval

The study adhered to the tenets of the declaration of Helsinki and was approved by the Ethics Commission of the Medical Faculty of the University of Tuebingen **(**reference 042/2018BO2**)** (https://www.medizin.uni-tuebingen.de/Forschung/Ethik_Kommission/Mitglieder+der+Ethik_Kommission.html).

### Data availabilty

The software for realtime analysis on the visual environment in terms of ON and OFF input strength can be downloaded at https://www.dropbox.com/s/t4um64aq6cjxoq3/ON20OFF20analysis20visual20world.zip?dl=0

Full data access (Excell file with complete data analysis) is available at https://www.dropbox.com/s/1pkh91eoh6ibwk7/Carillo20et20al20raw20data20Jan20162C202018.xlsx?dl=0.
